# The journey with dementia from the perspective of bereaved family caregivers: a qualitative descriptive study

**DOI:** 10.1186/s12912-014-0042-x

**Published:** 2014-11-27

**Authors:** Shelley C Peacock, Karon Hammond-Collins, Dorothy A Forbes

**Affiliations:** College of Nursing, University of Saskatchewan, Health Sciences Building, E-wing – 4340, 104 Clinic Place, Saskatoon, S7N 2Z4 SK Canada; 406-431 3rd Avenue North, Saskatoon, S7K 4Z3 SK Canada; Faculty of Nursing, University of Alberta, Level 3, Edmonton Clinic Health Academy, 11405-87 Avenue, Edmonton, T6G 1C9 AB Canada

**Keywords:** Bereaved, Dementia, Family caregivers, Journey, Qualitative description

## Abstract

**Background:**

With increasing rates of dementia among older adults, many people will be affected by this disease; either by having the disease or by caring for a relative with dementia. Due to a shift toward home and community-based care there will be an increase in the number of family caregivers caring for persons with dementia. The caregiving experience in the dementia journey is influenced by many factors. Currently there is a paucity of research that examines the dementia caregiving experience from the perspective of bereaved caregivers or that presents the complete caregiving journey. The purpose of this study was to describe the dementia caregiving journey as revealed by bereaved family caregivers.

**Methods:**

This study utilized qualitative description to describe the overall dementia caregiving journey as told by 11 bereaved caregivers. Open-ended interviews resulted in rich detailed descriptions of the caregiving journey from before a dementia diagnosis and into bereavement.

**Results:**

Findings are discussed based on the following caregiving themes: (a) getting a diagnosis; (b) managing at home; (c) transition to long-term care; (d) end of life; and (e) grief in bereavement. Subthemes reflect the dementia caregiving journey using the words of the participants. Participants spoke of grieving throughout the caregiving experience.

**Conclusions:**

Bereaved caregivers have similar experiences to active caregivers over comparable points in the journey with dementia. Findings from this work contribute new understanding to the literature around the unique perspective of bereaved caregivers, while presenting the overall dementia caregiving journey.

## Background

With increasing rates of dementia among older adult populations, a larger segment of society will be affected by this disease; either by having the disease themselves or by caring for a relative with dementia. Dementia is an umbrella term used to describe a range of cognitive disorders consisting of impaired memory, language and motor activity [1]. Increasing rates of dementia is a concern not only in North America but elsewhere in the world; the World Health Organization [2] estimates that the number of persons with dementia (PWD) worldwide is expected to double every twenty years. Due to a shift toward home and community-based care, it is anticipated that there will be an increase in the number of family or informal caregivers caring for PWD [3]. Currently, Canadians provide 15 million hours of informal care, by 2038 it is estimated that Canadians will provide 756 million hours of informal care for PWD. In 2010, the cost of dementia worldwide was estimated to be close to $604 billion USD [2]. It is clear then, that the need for family or informal caregivers is on the rise, as is the need to better understand and support caregivers in order to minimize the strain they experience and improve the care provided to PWD.

Research reveals that family caregivers may engage in caregiving out of a sense of responsibility, a desire to provide care or because no one else is available to provide care [4]. Some emotions experienced by family caregivers during the transition to becoming a caregiver include resentment, grief, frustration and a decreased relationship with the care receiver [5]. There is a strong need for caregivers and their relative to be supported from diagnosis onward [6]. Unfortunately, although it is a much-needed role, family caregiving has been associated with negative outcomes such as social isolation, depression, emotional and physical strain, poor physical health and decreased ability to work [7-9]. While much of the research focuses on burden and other negative aspects of family caregiving, positive aspects of caregiving do exist [10,11]. Some examples of positive aspects of family caregiving include a sense of self-efficacy, feeling of accomplishment, a sense of meaning, satisfaction, well-being and improved quality of relationships [12]. These positive experiences may help sustain family members in their work as caregivers [11].

The overarching goal of family caregivers and the healthcare system is to prevent or delay institutionalization of PWD, yet to achieve this there is a need for preventive formal supports for family caregivers [3,13]. Despite the family caregiver’s wishes to keep their relative at home, there comes a time in most caregivers’ journeys when the burden of care is too heavy and long-term care (LTC) placement is inevitable [14]. As a result, at some point in the caregiving journey, the PWD’s care needs may exceed the family caregiver’s capacity to provide care. Once the PWD is placed in LTC, the family caregiver has to trust others with their relative’s care, which can lead to positive or negative experiences [15]. Family caregivers often continue to provide care to their institutionalized relative, and though there is some relief and improved well-being among caregivers, depressive symptoms, anxiety and strain do not always decrease after placement [16].

The end-of-life stage of the journey with dementia, though equally important in the family caregiving experience, is also one of the least studied stages [17]. A review of the literature around this phase revealed grief, loss, burden, guilt, and depression were common experiences for family caregivers [18]. It was also found that family caregivers appreciated regular contact and connection with the healthcare professionals caring for their relative at the end of life. Another study describes the difficulty of watching a loved one fade away towards the end of life, that death still came as a shock to many family caregivers and that there may be a need for more information about the dying process to be made available to caregivers [15]. Consulting family caregivers about end-of-life care preferences for their relative and building supportive relationships with staff can have a positive impact on the caregiving experience during end of life [19].

Just as the end-of-life experience is understudied so too is the bereavement phase of the dementia caregiving journey. Chan, Livingston, Jones, and Sampson [20] conducted a systematic review regarding grief reactions in dementia caregivers and found of the three studies that examined bereaved caregivers, spouses are among those who experience severe or complicated grief owing to the prolonged period of caregiving and accompanying stress when their spouse finally dies. Further, greater amounts of grief experienced by family caregivers are related to higher levels of pre-death depression [21]. It is important to note that grief is a normal and expected reaction to the death of a loved one; the concern is with those who experience unresolved, complicated grief.

Additional quantitative research demonstrates that bereaved family caregivers to PWD experience significantly more depressive symptoms compared to non-caregiving groups [22,23]. Some studies reveal a decrease in depressive symptoms as bereavement progresses [16,24], while others note an increase in depressive symptoms [25]. On the other hand, a Spanish study conducted by Crespo, Piccini and Bernaldo-de-Quiros [26] found that among their sample of spousal and adult children caregivers, most (over 65%) did not develop any significant depressive symptoms over the study’s three year period. It is evident that dementia caregivers do not have uniform emotional responses to bereavement. This lends to the conclusion that the caregiving journey with dementia is enormously complex and that it does not end with the death of the PWD.

Bereaved family caregivers can offer unique insights into the caregiving journey and its multiple phases and are a valuable resource for better understanding the needs and experiences of family caregivers of PWD. Bereavement marks the final stage of their journey with dementia for family caregivers, adding another dimension to their reflections on all that their caregiving entailed and their most significant experiences. Yet the bereaved perspective is currently lacking in the research literature. Further, existing dementia research presents findings related to specific points in the caregiving journey (e.g., challenges in obtaining a diagnosis or decision making in the LTC setting), rather than presenting the overall journey. We could find no research that qualitatively describes the overall dementia caregiving journey from the perspective of bereaved caregivers. Thus, the purpose of this study is to add further clarity to the journey with dementia by describing the experience of caring for a relative with dementia from pre-diagnosis to bereavement, from the unique perspective of bereaved family caregivers. Thus, by using data collected from bereaved dementia caregivers, the research question that drives this work is: *What is the experience of caring for a relative over the long journey with dementia?*

The data utilized was originally collected for an interpretive phenomenological study conducted with 11 bereaved dementia caregivers [27]. This initial study explored the meaning of providing end-of-life care to a relative with advanced dementia and identified *being-with* and *being-there* for a dying relative as the essence of the end-of-life caregiving experience. The study also revealed the significance and complexities of various relationships for those providing end-of-life care in a long-term care home. Multiple interviews with the participants resulted in rich and detailed narratives that revealed far more, prompting this second study which analyzes the transcripts in order to describe the entire dementia caregiving journey, including bereavement.

## Methods

In order to describe the phenomenon of the overall dementia caregiving journey, qualitative description [28,29] was used to complete further analysis of open-ended interviews conducted with bereaved caregivers of PWD. Qualitative description aims to provide a straight description of phenomena in everyday language [28]. Although not free from interpretation, qualitative description attempts to make as few inferences as possible during data analysis [28,29]. A *phenomenological overtone* to the qualitative description was incorporated such that the lived experiences of caregivers caring for a relative from pre-diagnosis to bereavement were explored [29].

### Sample

Participants in the original study were recruited and interviewed between January and October 2010 by the primary author. Participants were purposively sampled because they were bereaved caregivers of a relative with advanced dementia who had passed away in the last three to twelve months. Rather than approach them as they were actively providing end-of-life care (and potentially interrupting their caregiving experiences), the participants were asked to provide a retrospective account of their end-of-life care once their relative had passed away. The participants included four wives, three husbands, three adult daughters, and one adult son who provided care to their relative for an average of six years. Spouses’ ages ranged from 65 to 89 years old (*M* = 79.3, *SD* = 7.5) and children’s ages ranged from 49 to 63 years old (*M* = 53.5, *SD* = 5.6). The care receivers’ ages ranged from 63 to 89 years (*M* = 81.5, *SD* = 7.0) and all had a confirmed diagnosis of some form of dementia and died in a LTC home.

### Data collection

During the original study, a total of 27 interviews from eleven participants were collected; each participant provided two interviews, while three participants each agreed to provide a third interview. All but one participant gave consent to have the interviews tape-recorded. The first interview began with a broad question: *What was it like to care for your dying [relative]?* Participants were not prompted about where or how to begin their story. Second and third interviews allowed participants to make additional comments and to check that the end-of-life caregiving story had been captured accurately. Despite the original study’s focus on the meaning of end-of-life care, the resulting rich and detailed interviews described many aspects of the caregiving journey from pre-diagnosis to bereavement calling for further analysis of the data. Hence this second study to analyze the data in the 27 transcripts for the participants’ overall journey with dementia.

### Data analysis

The interview tapes were transcribed during the original study. During the present study, transcripts were re-verified with the recordings by the research assistant to immerse herself in the data (as she was not part of the original study). The first step in analysis was to complete an “open” reading of each transcript to obtain an overall impression of its content. Across the transcripts it was readily evident each participant discussed key transition points in their dementia caregiving journey, from situations leading to a diagnosis and moving through to bereavement. Thus, to remain close to the data [28], the following major themes were developed from the data: *getting a diagnosis*, *managing at home*, *transition to long-term care, end of life,* and *grief in bereavement*. In the second step of analysis, each interview transcript was then re-read with the main themes in mind and codes were generated. The codes were then gathered into subthemes (i.e., step 3) by the research assistant and managed by the use of NVivo. Subthemes were titled using the precise words of participants when possible in order to maintain credibility [30]. A subset of three interviews was independently coded by the principal investigator to verify the subthemes developed by the research assistant; subthemes were critically discussed until agreement was reached, with very few modifications required. Lastly, the co-investigator verified the subthemes and their descriptions to ensure the caregiving journey was being accurately described.

All researchers attempted to set aside preconceived ideas and to remain as neutral as possible during data analysis to maintain confirmability. In order to maintain descriptive and interpretive validity, the findings from subthemes identified in ten or more interviews (i.e., once saturation of data was reached) will be presented in the findings section below as a descriptive summary of the data [28,29]. In order to maintain credibility and transferability of the findings of the present study, a bereaved caregiver who was not part of the original study was asked to read the findings section; this caregiver agreed with the content of the summary and felt that it resonated with her own dementia caregiving journey experience. This was done to determine if the descriptions resonated with the caregiver and if the researchers’ analysis was realistic and reasonable.

### Ethical considerations

In the original study, written informed consent was obtained from each participant at the beginning of the initial interview after being given information about the study and informed that they could withdraw at any time; second and third interviews obtained verbal consent and participants were again reminded they could withdraw from the study if they wished. The present study was approved by the Research Ethics Board of the University of Saskatchewan, Saskatoon, Canada (Beh #12-89) and followed the ethical standards set out by the Research Ethics Board. In order to protect the anonymity of participants, pseudonyms are assigned to participants and will be used in the presentation of findings.

## Results

Participants described many elements of their caregiving that resulted in a powerful and rich source for understanding the journey with dementia from the time prior to diagnosis and into bereavement. Findings are presented by utilizing the major themes of: (a) *getting a diagnosis*, (b) *managing at home*, (c) *transitioning to long-term care*, (d) *end of life,* and (e) *grief in bereavement.* Verbatim excerpts from the interviews are presented in data example tables to support the resulting subthemes for each category.

### Getting a diagnosis

When asked to share their experience of caring for a relative with dementia, six of the eleven participants began their narrative by describing the first indications of dementia they noticed or the diagnosis they received. Participants described these ‘first signs’ of dementia they noticed, such as forgetting ingredients in a recipe. They also spoke about the process of receiving a diagnosis and the ways they ‘prepared for the future’ such as moving to safer accommodations and preparing legal matters. Lastly, the theme of ‘accepting the next step’ emerged in which participants described the tragic losses associated with dementia experienced by both the caregiver and care receiver. Please see Table 1 for data examples of the subthemes for getting a diagnosis.

**Table 1:** **Getting a diagnosis**

**Subtheme**	**Data**
**First signs**	“I didn’t realize at the time something was – I knew she was different, but I thought well, she’s just getting older, maybe that’s the way it is, but I realized it later on that year where something was different than I was thinking.” ~ Rudy
	“Well, preceding that she was having memory problems. For example, she liked to bake, she was a good cook. And she would make a recipe, a fairly familiar one and it wouldn’t turn out and of course that recipe resulted from her leaving out something that was essential.” ~ Dale
**Diagnosis**	“It took, well, took two years of testing. They did every test in the book to try and figure out, well to try to find if it was something else, and everything else showed that it was fine. We had to come to grips with Alzheimer’s and he was diagnosed.” ~ Lois
**Preparing**	“That is something, mind you, as far as funeral arrangements we decided together… Helen and I used to talk a lot and discuss different situations so we had a pretty good feeling of what we wanted, so we set it up that way. ” ~Rudy
	“So what we did is we dealt with doctors and healthcare workers, CPAS [Client Patient Access Services], lawyers, accountants, those types of things; we were pro-active rather than reactive. What we tried to do is ensure that there was care and protection for her for her safety…” ~ Laurie
**Accepting the next step**	“Every so often I would catch him sitting with a little tear in his eyes *{she points to the corner of her eye and drags her finger down her cheek}.* That was when I knew he had a moment of clarity that he saw the future and he knew how much he had lost.” ~ Lois
	“I’d say, ‘Okay, what can we do, I can’t lift you’, ‘Oh, just get me a bottle of aspirins… so I was angry he wasn’t facing the future… He was so angry, he just could not grasp this next step, and it was evident to me the whole time long that, that he could not grasp that, and the pills had nothing to do with dying at that moment, it had to do with not going to the nursing home, and I knew that.” ~ Alice

### Managing at home

Participants discussed at length the activities of caregiving they performed while the PWD was still at home. Much of the support they provided related to helping the care receiver to function on a daily basis. Through the many challenges they discussed, participants also noted that they were able to find the strength needed to provide care to their relative. Participants provided a great deal of insight into the trajectory of dementia, as they were able to observe little bits of their relative disappearing over time as they succumbed to the effects of dementia. Please see Table 2 for data examples of the subthemes for managing at home.

**Table 2:** **Managing at home**

**Subtheme**	**Data**
**Caring at home**	“We had to get the equipment in so he could get out of the bathtub and get up from the toilet and he could manage in the house. He was, he was very, very incontinent. It was a matter of changing depends a lot every day.” ~ Jane
	“But by and large looking back over it, it wasn’t too bad. One of the worst parts of the thing was when she was wandering around the house at night and trying to get outside on occasion. That was a trouble for me… You can be asleep but still aware of everything that’s going on and you kind of develop that sense.” ~ Dale
**Found strength**	“But you learn an awful lot and you learn what you can do. What you can do I had no idea I would be able to do some things. It’s like having a toddler you think ‘Oh my God I couldn’t change a child’s diaper.’ But it is your child and you do it. And it’s different - it’s your person and you do what has to be done because you don’t want them to be uncomfortable…” ~ Lois
	“I really had doubts at first when all this happened that if I would be able to do this sort of thing, right?” ~ Charles
**Little bits disappearing**	“He just disappeared on me. That was the Paul of then. My Paul had gone years before. And had just kind of… little bits just disappeared over that seven-year period.” ~ Lois
	“She put her tablets together in her pillbox, okay? She needed to know which of the drugs were at two o’clock, this one in the morning; this one was at night time. She started to get these mixed up a little bit. And wow, that’s probably when it, we had our biggest problem. She didn’t want to give this up because, “I’m the pharmacist of thirty-three years and I know what I’m doing!” It was her last bit of independence, right?” ~ Charles

### Transitioning to long-term care

Participants shared many details about their experiences of transferring their relative to LTC and the process of caring for them in their respective facilities. The decision to transfer their relative to LTC usually began with a ‘tipping point’ of some kind, which made the caregiver realize that continuing to provide care at home was no longer a viable option. Then participants discussed how they and the PWD were integrated into the facility, sharing their positive and negative experiences while in LTC. Participants also explained how the care they provided changed or remained the same after the transfer to LTC. Please see Table 3 for data examples of the subthemes for transitioning to LTC.

**Table 3:** **Transitioning to long-term care**

**Subtheme**	**Data**
**Tipping point**	“I tried to get home care to come in and they said unless he was willing to cooperate - that was the word, this wonderful word - if he wasn’t willing to cooperate, they couldn’t come in and help me…I thought that was the tipping point and I knew at that point that he needed to go to the program or rather into long-term care.” ~ Lois
	“And I don’t know what triggered putting him in a nursing home. I think, it was that my two kids thought I wasn’t going to last. So we put him in the first care home.” ~ Jane
	“It ended up my kids took me to Mexico, so I had to put Helen in at [name of LTC facility] just for the time I was away… but when I came back she had fallen and couldn’t walk so I couldn’t take her home, so it was decided then that she would maybe stay there which she did.” ~ Rudy
**Hardest day**	“Like the day I had to sign the papers to put her in [facility]. That was my hardest day. You know, that was the day that she wasn’t coming back home. That was the last time… That was the hardest.” ~ Charles
	“…All the while you’re lying, right, you had to tell them it was the best thing for them, so…but you’re trying to, you know, remove myself as much as you could, but yet knowing that…so it’s just really hard as you can imagine… And I realized my end was coming of caregiving for Dad, knowing that the time in that home for Dad would be very different. Dad would change, I would change. ~Tom
**Integrating into facility**	“But, on the other hand, the staff they tried their very, very best to make it—not like my home, but kind of a composite home and they tried very hard to make it welcoming and, and inclusive for all of the people including the caregivers that were there.” ~ Lois
	“In order to comfort my mom in some instances some of the caregivers would actually lie to her and tell her that she’d better get her coat on because I’m coming to get her and they’d make her stand at the top of the stairs and wait for me to come which I never knew I was going to be coming, so that was really sad. The training was lacking in some of the caregivers, not all, but in a few it was really, really sad.” ~ Laurie
**Caring in LTC**	“I very much felt that I was part of the staff. I felt that they really needed the caregivers to continue to do a lot for the people, again, because their staff was so short… You know, if you would go and you would help feed your person, that was huge for them and they really did appreciate it … and I mean, a lot of caregivers want to do it anyway, so it was a win-win situation.” ~ Lois
	“I went in the morning, you know, came home for lunch, and would go back again around before supper time so I could help her you know, feed her and this sort of thing. If she would eat.” ~ Charles

### End of life

Regarding the participants’ experiences caring for their relative toward the end of life and into the dying process, several comments were made that related to the way caregivers made decisions. They also spoke about how much warning they received about the death of their relative, if any. Participants shared their observations of the PWD ‘slipping away’ before death, what it looked and sounded like to them. Several participants explained that, although sad, their relative’s death was also the ‘best thing’ for their relative because it meant an end to their suffering. Reflections were made about the overall caregiving journey and what meaning caregivers were able to derive from it. Finally, some thoughts were shared about the process of moving on after the death of their relative. Please see Table 4 for data examples of the subthemes for end of life.

**Table 4:** **End of life**

**Subtheme**	**Data**
**Decision-making**	“You’re taking somebody else’s life and making these decisions. So that’s the kind of suffering that I went through.” ~ Lois
	“I emphasize ‘her wishes’ because it’s very important for people that are dealing with parents to realize that if you’re dealing with Alzheimer’s or dementia you soon become the parent, you become the caregiver of these people, they are no longer your look-to person, they are the person who’s looking to you… If you prepare, the death and dying process is manageable, but if you’re not prepared then you run into other obstacles which I don’t know might not be the most fun.” ~ Laurie
**Warning**	“The nurse in charge, phoned and said, ‘I think the end is coming very close.’ She said, ‘her breathing is getting pretty shallow’ and this sort of thing, ‘you might want to come up.’ And I came up to stay with her.” ~ Charles
	[Mom] knew she was going to die… She even breathed really weird, for probably four or five days, like where she would, umm, breath really… She would hold her breath, how she would hold her breath for 20 seconds and then all of a sudden let out a heavy sigh, then hold it. So she did this for a long, long time. So we had lots of warning.” ~ Lenoa
**Slipping and passing away**	“Soon after his brother left he, it was almost as if he just kind of forgot how to swallow and he could chew and chew but he didn’t know what to do. And then he kind of stopped eating and he just kind of slipped away. He just disappeared on me.” ~ Lois
	“Then she finally took the last breath. But we just kept saying, ‘Mom, it’s ok, we won’t be sad, you’ll be happy you’re in heaven.’ We played ‘How great thou Art’ and she finally just relaxed and went to sleep. I felt peace…” ~ Lenoa
**Best thing**	“I was just with him. I dozed off and I woke up and he was just gone. And that was the best thing. I couldn’t wish him back, not to the way he was, you know.” ~ Lois
	“And, and so I thought well, you know, he wanted to die. When I got the call … we all went to the nursing home together. He was lying there peaceful and I said, ‘Hey, you did it. Good for you,’ you know, ‘Hey…you did it. Good for you.’ And, and so I couldn’t wish anything better for him than to die peaceful in his sleep.” ~ Alice
**Meaning**	“It was all I could do, so you know I felt… I was glad that I could do it. I mean, you know, that’s all you could do. You know when you think about your parents, how much they did for you over your lifetime, so I was very happy to do it.” ~ Claire
	“The gift of giving to him and caring is so mutual… So, you know, I just feel very blessed by it and happy that I had the time to do it and the health to do it and my family had the health and to do it and the wealth to do it.” ~ Tom

### Grief in bereavement

Beyond the major stages of caregiving explored in this study, many participants discussed their process of grieving during their interviews. Some comments related to ‘grieving the relationship’ the caregiver had had with the care receiver. Other comments pertained to ‘grieving the person before dementia’ where participants reflected their memories of their relative prior to the dementia journey. Some participants even felt their relative had ‘died a long’ time ago, before their physical death, due to the effects of dementia. After their bereavement, caregivers recounted that they grieved their relative with dementia and, by extension, the caregiving role they used to fill. Please see Table 5 for data examples of the subthemes for grief in bereavement.

**Table 5:** **Grief in bereavement**

**Subtheme**	**Data**
**Grieving relationship**	“You plan these long-term things that you can do and then you realize you’re going to be doing it by yourself and that’s not what you bought into. And there’s a lot of anger. There’s a lot of anger. And I still get very angry with him for leaving me. Then you say ‘Hey, it wasn’t his choice’ that’s for sure.” ~ Lois
	“I think the hardest part is I just miss talking to her. She was a very supportive mother and I really missed that. We would still visit with her, we would have a meal together and we would go to their place or she and dad would come over. And I just really miss talking to her and sharing what is going on with my life. That’s one thing for me personally.” ~ Claire
**Grieving person before dementia**	“Just at the anniversary of his death I realized for the first time I had stopped grieving the person that died, I had started to grieve for the person he was before he got sick. It was really, it was kind of a break point there and maybe one forgets the person that was struggling and suffering and that you had to do all these things for and remember the person you married.” ~ Lois
	‘She states that now, months later she is beginning to grieve the old Bob, the good Bob, the Bob of their good years together. She has done enough grieving of the Bob of the last three years, the dementia Bob. Now it feels good to remember all the good times and look at the happy picture of the two of them, smiling and laughing together.’ - notes from an interview with Rose
**Died a long time ago**	“I didn’t feel like she was my Mom for a long time…. It’s more than the 10 to 11-year trauma we went through as we lost Mom slowly over ten years than it was the actual death, because the death we were ready for.” ~ Leona
	“So I lost her quite some time ago. I lost her on May the 3rd, but I lost my mom and the connection, that type of connection I had, mother-daughter, that we had a long time ago.” ~ Laurie
**Grieving person with dementia**	“Different kinds of grieving over a period of time. And when he first died it was the person that I went to see every day and to feed and to interact with the other people in that community. It was that person and that was gradually, gradually slipping away. ~Lois

## Discussion

This paper provides further understanding of the long journey with dementia using the insights and experiences of bereaved caregivers. This is important as bereaved caregivers have the advantage of reflecting back on their dementia journey without being influenced by the demands of providing care in the moment. Much of the discussion that follows is completed with studies that include non-bereaved caregivers given the paucity of research that examines the dementia journey from the bereaved caregiver’s perspective.

The descriptions of ‘first signs’ of dementia in this study are corroborated in a study by Davies [31], which found that participants at first tried to normalize changes in the care receiver’s behaviour before having to accept that ‘something’s going on’. Despite data regarding the length of time it took to receive a diagnosis was not specifically collected, participants tended to describe getting a diagnosis as a process which most likely would have taken place over a lengthy period of time, as found in other research which described this process as a diagnostic journey [14,32]. Although caregivers in other studies have been found to be poorly prepared to assume the role of a caregiver [33], participants in the present study discussed their attempts to prepare for the future needs of their relative and did not necessarily express feeling ill-prepared.

Participants described how they and the care receiver dealt with accepting the progression of losses due to dementia, such as recognizing the trajectory of the disease and the associated cognitive decline. Similar themes of disruption and loss were found by Boylstein and Hayes [34]. Doka [35] also found that feelings of grief can arise when the anticipated symptoms and losses due to dementia are acknowledged. These losses may pertain to changes in relationship, roles, independence, memory and self [35]. Moreover, Garand et al. [36] suggest that caregivers of newly diagnosed PWD would benefit from interventions early on to address the many losses associated with dementia and support caregivers to cope with anticipatory grief.

Participants described both positive and negative aspects of their caregiving at home. Some of the negative experiences described by caregivers include fatigue, difficulties with activities of daily living and caring for a person with incontinence. Physical, emotional and financial stressors as well as being overwhelmed with care are often cited in the literature and support the experiences of caregivers in this study [14,37]. Not all participants had negative experiences as some described their caregiving at home as being a learning opportunity. Others refer to it as being ‘not too bad’ and later go on to express regret about the transition to LTC. One caregiver shared that his wife was able to express gratitude for the care he was providing to her. Some factors that have been identified as contributing to positive experiences with caregiving include a positive marital relationship [38] as well as the ability to find pleasure or purpose in caregiving [39]. Other studies have found that up to 81% of caregiver participants reported both strains and gains from their caregiving experience, such as multiple demands, uncertainties and fatigue on the one hand and personal growth and mastery on the other [37].

During this period of caregiving, participants conveyed at length the physical, emotional and social losses they experienced as a result of the progression of their relatives’ dementia. They described this process as ‘losing the person’ and watching them disappear over a period of time. Boylstein and Hayes [34] described the losses that arise with worsening dementia, and which have consequences for the family caregiver such as social isolation and changing roles and relationships. Other types of loss experienced by the PWD documented by Doka [35] include loss of personality, independence and cognitive functioning, which can lead to a decrease in intimacy and companionship in family caregivers. Other expressions that are used to describe these losses include ‘social death’ and ‘former selves’ [40] which support the feelings experienced by caregivers in this study that their relative had disappeared.

In this study, several participants referred to a ‘tipping point’ in their caregiving where they realized that transfer to LTC was necessary. Other studies have also found that the growing needs of PWD *tipped the scale* in favor of placement in LTC or that a crisis point triggered the need for additional care [41,42]. In some cases, caregivers perceive LTC to be an unacceptable alternative to caring at home, but have no other means to meet the needs of their relative [43]. Participants described the move to LTC as being their ‘hardest day’ and many of them expressed doubts about the transition. Although caregivers may experience relief after placement, feelings of sadness, loss, guilt and betrayal are not uncommon in caregivers during this transition [41,43].

Once in LTC, some caregivers continued to provide care to their relative. Some participants described that they wanted and needed to visit and care for their relative. The transition from caring at home to caring in LTC can be challenging and many caregivers may want to be perceived as the primary carer and have a presence in LTC [15,42,44]. A study by Duggleby, Schroader and Nekolaichuk [45] found that dementia caregivers desire a continued connection to their relative in LTC and that the relational aspects of hope are essential and important to that relationship. These findings indicate that the caregiving journey entails new and different responsibilities after the PWD transitions from home to LTC. Moreover, this specific stage necessitates dramatic shifts in roles and routines for both the family caregiver and the PWD, and their need for support may be especially high at this time. Healthcare professionals/providers should take into consideration the profound changes occurring in the lives of family caregivers and provide support appropriately during these transitions.

When describing the decision-making processes around end of life, family caregivers highlighted how distressing it can be to make decisions for another person, that one may have to assume a parental role to the PWD, and that preparing for the end of life is vital. These findings are corroborated by other studies that similarly found high information needs among caregivers [46]. Participants in a study by Waldrop and Kusmaul [47] also described *exquisite discomfort* related to making decisions for their relatives. Lastly, health care professionals/providers’ communication with family caregivers and involving them in decision-making can have an impact on their satisfaction with the care provided [48].

Participants had varying amounts of warning about the nearness of their relative’s death. Some had time to gather with family for several days to say their farewell, while others had no indication from staff or otherwise that death would come when it did. Other research has found that many family caregivers are shocked when death occurs and that further information about the dying process may need to be provided to caregivers [15]. It may also be that the staff do not themselves recognize that the PWD is dying and therefore the family is not informed when the time approaches [47].

In the period immediately before death, participants described their relative as ‘disappearing’. The actual death of the PWD was often described as falling asleep or simply that they stopped breathing, and were perceived as having a peaceful transition. Participants acknowledged they were glad to see an end to their relative’s suffering, but some also did not want to let their relative go. During this final stage, healthcare professionals/providers should take the opportunity to inquire about family preferences and assist with end-of-life decision making ahead of time. Preparation for the end of life, as emphasized by participants, makes this time easier to manage.

Experiences of grief throughout the caregiving journey were shared by many participants. One element of grief that was recounted related to grieving the relationship the participants had with the PWD. Personality changes and cognitive deterioration in the PWD may contribute to changes and losses in the relationships between caregiver and care receiver [35]. Caregivers in other studies were found to hold on to traces of their relative that were still familiar to them, while acknowledging that their relationship was drastically changed: for example a wife comparing caring for her husband to having a child instead [40]. In addition to changing relationships, some participants commented on the grief they experienced losing the person they knew before the dementia set in, even describing it as a death of their relative who was ‘lost’ to them a long time ago. These descriptions appear in other research, where participants described their relative with dementia as *having gone* and using the past tense when they refer to their still-living relative [40]. It is important for healthcare professionals/providers to be aware that throughout the caregiving journey, the relationship family caregivers have with their relative may change and this may have an impact on their level of motivation and satisfaction related to the caregiving role.

Finally, family caregivers in this study discussed that following the death of their relative, they missed the person they had cared for and grieved the PWD. This experience is not uncommon, as family caregivers can grieve the end of their caregiving role [35]. This type of grief may not always be socially sanctioned, due to the fact that others may expect the caregiver to feel relieved and liberated after the death of the PWD, and is sometimes termed *disenfranchised grief* [35]. It is important to note that grief changes over the course of bereavement, and while such changes could not be fully explored in the present study, it nonetheless warrants further investigation. Healthcare professionals/providers must take care not to assume how a bereaved caregiver feels about their changed role, but rather to explore it with them in order to tailor the support that is needed.

### Implications for practice and future research

Healthcare professionals/providers who interact with family caregivers of PWD need to appreciate that there are many stages to the caregiving journey and that each of these stages presents unique challenges and stressors to the family caregiver. Family caregivers’ experiences will vary depending on their relationship to the PWD, the stage of the caregiving journey they are in, as well as their unique coping styles and available supports. Healthcare professionals/providers should be aware that caregiving can be burdensome but also has many affirmative aspects and should inquire about the family caregiver’s positive and negative experiences. Some areas for future research include examining differences in caregiving experiences between children and spouses of PWD, as well as the gender differences in caregiving experiences.

### Limitations

Although the sample size was small and may limit the transferability of the findings, qualitative study sample sizes are usually small due to the large volume of narrative data to be analyzed [30]. The data for the present study was originally collected to explore the end-of-life caregiving experience, not to focus on describing the dementia caregiving journey. Further, all interviews were conducted with caregivers who were recently bereaved (less than 12 months) and may have still been actively grieving their relative; in other words, the retrospective approach to the participants’ reflections may have influenced their recollections of the dementia caregiving journey. Other limitations to external validity and generalizability of findings to other groups may exist due to sampling techniques; however, efforts were made to verify findings with a family caregiver external to the study to determine if the results of this study resonated with her experience.

## Conclusions

To the best of our knowledge, this is the first study to present the overall dementia caregiving journey from the perspective of bereaved caregivers. The findings of this study support findings present in other studies using *active* caregivers at particular points in the caregiving journey and also contribute new learning to the literature around the unique perspective of bereaved caregivers. Participants tended to describe their journey with dementia in a manner that fit with the major categories of getting a diagnosis, managing at home, transitioning to LTC, end of life, and grief in bereavement. Another theme that emerged repeatedly was the grieving process throughout the caregiving experience. This data provides valuable insights in the experiences of bereaved caregivers even following the death of their relative, which can help to bridge the gap in the current literature related to the end-of-life experiences of caregivers. Health care professionals/providers can benefit from a better understanding of the journey that bereaved caregivers of PWD go through in order to provide appropriate support to the family caregivers they encounter. This study demonstrates that each stage of their entire journey with dementia has its own significance to family caregivers. Equally important, this study shows that health care professionals/providers must be cognizant of the journey’s complexities as they interact with family caregivers.

## Abbreviations

LTC: Long-term care
PWD: Persons with dementia
